# Rare disease variant curation from literature: assessing gaps with creatine transport deficiency in focus

**DOI:** 10.1186/s12864-023-09561-5

**Published:** 2023-08-16

**Authors:** Erica L. Lyons, Daniel Watson, Mohammad S. Alodadi, Sharie J. Haugabook, Gregory J. Tawa, Fady Hannah-Shmouni, Forbes D. Porter, Jack R. Collins, Elizabeth A. Ottinger, Uma S. Mudunuri

**Affiliations:** 1 Advanced Biomedical Computational Science, Frederick National Laboratory for Cancer Research, Frederick, MD 21702 USA; 2 Division of Preclinical Innovation, Therapeutic Development Branch, Therapeutics for Rare and Neglected Diseases (TRND) Program, National Center for Advancing Translational Sciences, National Institutes of Health, Bethesda, MD 20892 USA; 3 Division of Translational Research, Eunice Kennedy Shriver National Institute of Child Health and Human Development, National Institutes of Health, Bethesda, MD 20892 USA

**Keywords:** Rare disease, Gene variant, Literature curation, CTD, *SLC6A8*, Variant database, Text mining

## Abstract

**Background:**

Approximately 4–8% of the world suffers from a rare disease. Rare diseases are often difficult to diagnose, and many do not have approved therapies. Genetic sequencing has the potential to shorten the current diagnostic process, increase mechanistic understanding, and facilitate research on therapeutic approaches but is limited by the difficulty of novel variant pathogenicity interpretation and the communication of known causative variants. It is unknown how many published rare disease variants are currently accessible in the public domain.

**Results:**

This study investigated the translation of knowledge of variants reported in published manuscripts to publicly accessible variant databases. Variants, symptoms, biochemical assay results, and protein function from literature on the *SLC6A8* gene associated with X-linked Creatine Transporter Deficiency (CTD) were curated and reported as a highly annotated dataset of variants with clinical context and functional details. Variants were harmonized, their availability in existing variant databases was analyzed and pathogenicity assignments were compared with impact algorithm predictions. 24% of the pathogenic variants found in PubMed articles were not captured in any database used in this analysis while only 65% of the published variants received an accurate pathogenicity prediction from at least one impact prediction algorithm.

**Conclusions:**

Despite being published in the literature, pathogenicity data on patient variants may remain inaccessible for genetic diagnosis, therapeutic target identification, mechanistic understanding, or hypothesis generation. Clinical and functional details presented in the literature are important to make pathogenicity assessments. Impact predictions remain imperfect but are improving, especially for single nucleotide exonic variants, however such predictions are less accurate or unavailable for intronic and multi-nucleotide variants. Developing text mining workflows that use natural language processing for identifying diseases, genes and variants, along with impact prediction algorithms and integrating with details on clinical phenotypes and functional assessments might be a promising approach to scale literature mining of variants and assigning correct pathogenicity. The curated variants list created by this effort includes context details to improve any such efforts on variant curation for rare diseases.

**Supplementary Information:**

The online version contains supplementary material available at 10.1186/s12864-023-09561-5.

## Background

A rare disease is defined as a life-threatening or chronically debilitating disease that affects fewer than 200,000 people in the United States and fewer than 1 in 2,000 or 1 in 2,500 people in Europe or Japan, respectively [1]. Rare diseases may individually be rare but collectively are a common problem that have significant medical and societal impact. Rare diseases were estimated to have cost $1 trillion in the United States in 2019 when accounting for absenteeism, lost work production, and hiring caretakers [2]. Between 4 and 8% of the world’s population are affected by a rare disease at any point in time [3–5]. This includes approximately 20–30 million Americans, 46 million Europeans, and 470 million people worldwide [5]. This number does not include the many rare disease patients who do not survive infancy.

Historically, it has been difficult to diagnose rare disorders with a genetic etiology through phenotype or symptoms alone. Recent innovations in genome sequencing are leading to more rapid diagnosis and precise molecular-level characterization of rare diseases. Incorporating genome sequencing to identify causal variants has already shortened the diagnostic odyssey for many patients; in one study producing diagnoses for unsolved cases that had averaged 19 years since symptom-onset without a diagnosis [6]. However, utilizing genetic diagnosis requires either an extensive library of definitively classified variants, pathogenicity prediction algorithms with clinic level trustworthiness, or the logistics and funding to support an expert geneticist able to interpret newly discovered variants of uncertain significance (VUS) [7]. Pathogenicity information is valuable; each classification represents hours of expert labor. In order to classify novel variants, an expert needs to be able to review primary publications, call for biochemical assays to corroborate or refute the molecular diagnosis, and perform segregation studies on the family’s variant and phenotype inheritance [8]. This process of expert classification of variant pathogenicity is prohibitively expensive and expertise limited, putting it out of reach for many patients.

The National Organization for Rare Disorders (NORD) reported in 2021 that fewer than 10% of rare diseases have a treatment [9]. Some of these treatments target specific mechanisms of dysfunction, such as oligonucleotide induced alternative splicing [10] or employing a chaperone molecule to correct erroneous protein folding [11]. The genetic variant’s category of dysfunction can determine which interventions are possible. Recent FDA approval of therapies specifically targeting different classes of genetic variants for cystic fibrosis patients [12] exemplifies variant class based therapeutic approaches for treating rare diseases. A data set of rare disease variants and functional consequences could lead to shared insights about the mechanisms of action across multiple disease genes, and lead to the discovery of therapies that target multiple related diseases. Such a database could also be potentially used by protein structure modelling algorithms to programmatically identify the variant’s impact on protein structure and function, and its potential druggability. Access to collated data on genetic variants, their pathogenicity, and associated symptoms, is therefore vital for both rare disease diagnosis and research on therapeutic interventions.

There are currently well-known initiatives such as the ClinVar database [13] that allow researchers and clinicians to publicly share newly discovered variants and clinical associations with others in the field. Efforts have also been made to mine and share variant details found in literature. However, while literature curation and mining variants for specific diseases has long been recognized as essential for research, there are currently no open access databases containing all literature curated variants and their functional and clinical relevance for all rare diseases. While experts agree that it is critical to share and be able to access the known classifications for solved variants with indisputable pathogenicity classifications, it is not known if all the published pathogenic variants are easily findable for use in diagnostic panels or available for researchers to study functional and therapeutic significance.

This investigation set out to understand the gaps between variants published in literature and those accessible through open access data sources by manually curating and analyzing a dataset of all variants ever published for one rare disease from biomedical literature. The primary goal was to quantify data translation gaps between published variants with well documented pathogenicity details, and the variants available in publicly accessible databases. A secondary goal was to assess the accuracy of *in-*silico prediction algorithms at predicting pathogenicity of the published variants. One of the important aspects of the study was to collect extensive clinical and functional context details. Because it is not feasible to perform manual curation of published literature for the thousands of rare diseases, this data can be used to guide automation pipelines for scaling the effort. Curating clinical and functional details that contribute to pathogenicity assessments helps identify additional data that can be integrated into an automated literature curation workflow.

**Fig. 1:**
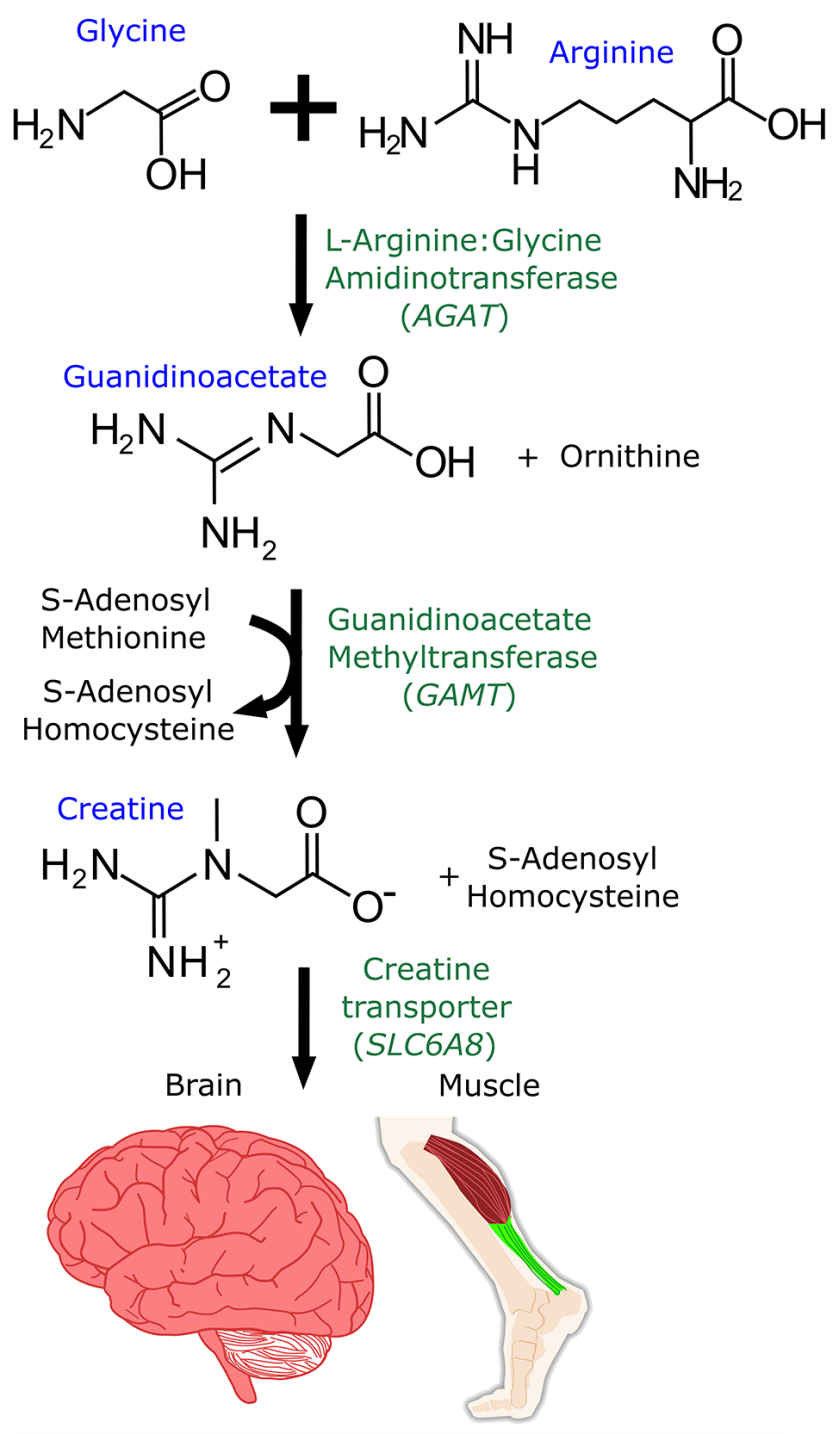
Creatine Synthesis. Creatine can be synthesized in cells or transported via the creatine transporter *SLC6A8*. Human metabolic synthesis of creatine from arginine and glycine via *AGAT* and *GAMT* is shown. Loss of function in *SLC6A8* causes X-linked Creatine Transport Deficiency (CTD).

X-linked Creatine Transporter Deficiency (CTD) (see Fig. 1) was chosen as an ideal candidate for manual curation because it is a monogenic disorder with a phenotype largely dependent upon the function of a single gene, *SLC6A8*, at a hemizygous location [14] on the X chromosome. CTD symptoms first present at approximately two years of age, a time shown to be too late for intervention [15] in other cerebral creatine deficiency disorders. Guanidinoacetate methyltransferase (*GAMT)* deficiency is a related disease caused by impaired cerebral creatine synthesis (Table 1). Oral supplementation of creatine started in *GAMT* patients younger than one month old leads to normal developmental outcomes, but when treatment is initiated later, there is some level of intellectual disability [15]. This models what an intervention to restore cerebral creatine could do for CTD patients, although one has not yet been developed. Unfortunately, one month old is before the symptom onset of CTD, so diagnosis dependent upon the appearance of first symptoms at two years of age might be too late for intervention. Whole genome sequencing is now more affordable than ever [16]. Combining newborn screening with the ability to rapidly and correctly interpret pathogenic *SLC6A8* variants has the potential to identify patients in infancy and allow intervention during the time window when the brain is still amenable to therapeutic intervention.

**Table 1:** Genetic Causes of Cerebral Creatine Deficiency Syndrome (CCDS). Insufficient import (*SLC6A8*) or synthesis (*AGAT*, *GAMT*) of creatine can cause CCDS, which results in low or absent cerebral creatine peak as measured by magnetic resonance spectroscopy (MRS).

Disease	Gene	Protein	Diagnostic Test	Treatment
Creatine Transport Deficiency	*SLC6A8*	Creatine transporter, Solute Carrier Family 6 (Neurotransmitter Transporter, Creatine), Member 8	Brain MRS lacks creatine peak. Males: Elevated urinary creatine:creatinine ratio relative to age matched controls. Females: DHPLC or fibroblast creatine uptake or D3 labeled creatine wash out assay.	No approved treatments. Potential therapies might include cyclocreatine and 4PBA.
AGAT Deficiency	*AGAT*	L-Arginine:Glycine Amidinotransferase (GATM)	Brain MRS lacks creatine peak. Plasma and urine GAA and creatine + creatinine added together are below normal range. Low urinary guanidinoacetate excretion (approximately 10% of control).	400 mg/kg dietary creatine monohydrate
GAMT Deficiency	*GAMT*	Guanidinoacetate Methyltransferase	Brain MRS lacks creatine peak. Low creatine abundance but elevated guanidinoacetate in plasma, urine, and cerebrospinal fluid. Creatine + creatinine added together are decreased in both plasma and urine.	Dietary creatine monohydrate, but guanidinoacetate remains elevated

CTD was first described in 2001 in a six year old developmentally disabled male patient whose proton magnetic resonance spectroscopy (MRS) revealed an absence of creatine in the brain [17]. The first pathogenic *SLC6A8* variant was found through sequence analysis of amplified cDNA from this patient’s fibroblasts [18]. The hemizygous inheritance pattern and clinical impact of the c.1540 C > T, p.R514X, HG38 chrX: NC_000023.11: 153,694,577 C > T variant in *SLC6A8* was documented in detail in the patient and relatives [19, 20]. In the two decades since the disease was described, researchers have published dozens of manuscripts on CTD patient symptoms [21–40] and classified the pathogenicity of many *SLC6A8* gene variants. *SLC6A8* also has a researcher submitted variant list available through the Leiden Open Variation Database (LOVD) [41], which can serve as a standard dataset for comparative analysis with the manually curated literature variants.

This study reviewed all published literature on CTD and *SLC6A8* as of Dec 1st, 2020. Variant mining from literature involved: finding the disease associated pathogenic and benign variants, harmonizing to remove duplicates, collecting phenotype, protein function and biochemical assay data, and assigning pathogenicity. A thorough analysis of the variants, their role in pathogenicity, and comparisons with the variant information in prominent clinical and population variant databases is included. In addition, pathogenicity predictions by variant impact algorithms, gaps uncovered through this exercise, and approaches for integrated and scalable processes to mine the information for other rare diseases are explored.

## Results

### CTD genomic variant analysis

The literature curation of published manuscripts and harmonization process to consolidate variants with multiple names resulted in a list of 185 unique published variants in *SLC6A8*. Harmonizing to genomic location revealed that multiple authors had published the same variant using different notations. Variants were published under multiple names because of the use of IVS notation method, such as IVS7-99 C > A and IVS12 + 32 C > A being the same as c.1142-98 C > A (HG38 chrX: NC_000023.11: 153,693,807 C > A) and c.1767 + 32 C > A (HG38 chrX: NC_000023.11: 153,694,921 C > A), respectively. The same intronic variant could be referred to as starting from the last base of the exon before the intron, or from the first base of the exon after the intron. Another source of differing notations was due to lack of standardization when writing duplicated nucleotides. For example, c.1016_41dupTGCCC and c.1016 + 41_45dupTGCCC were referring to the same variant but notated differently (HG38 chrX: NC_000023.11: 153,693,407 dup TGCCC). One variant was found to have been published using a non-canonical protein transcript reference: p.G351R had the same genomic position as p.G466R (HG38 chrX: NC_000023.11: 153,694,347 G > A) reported by other authors.

**Fig. 2:**
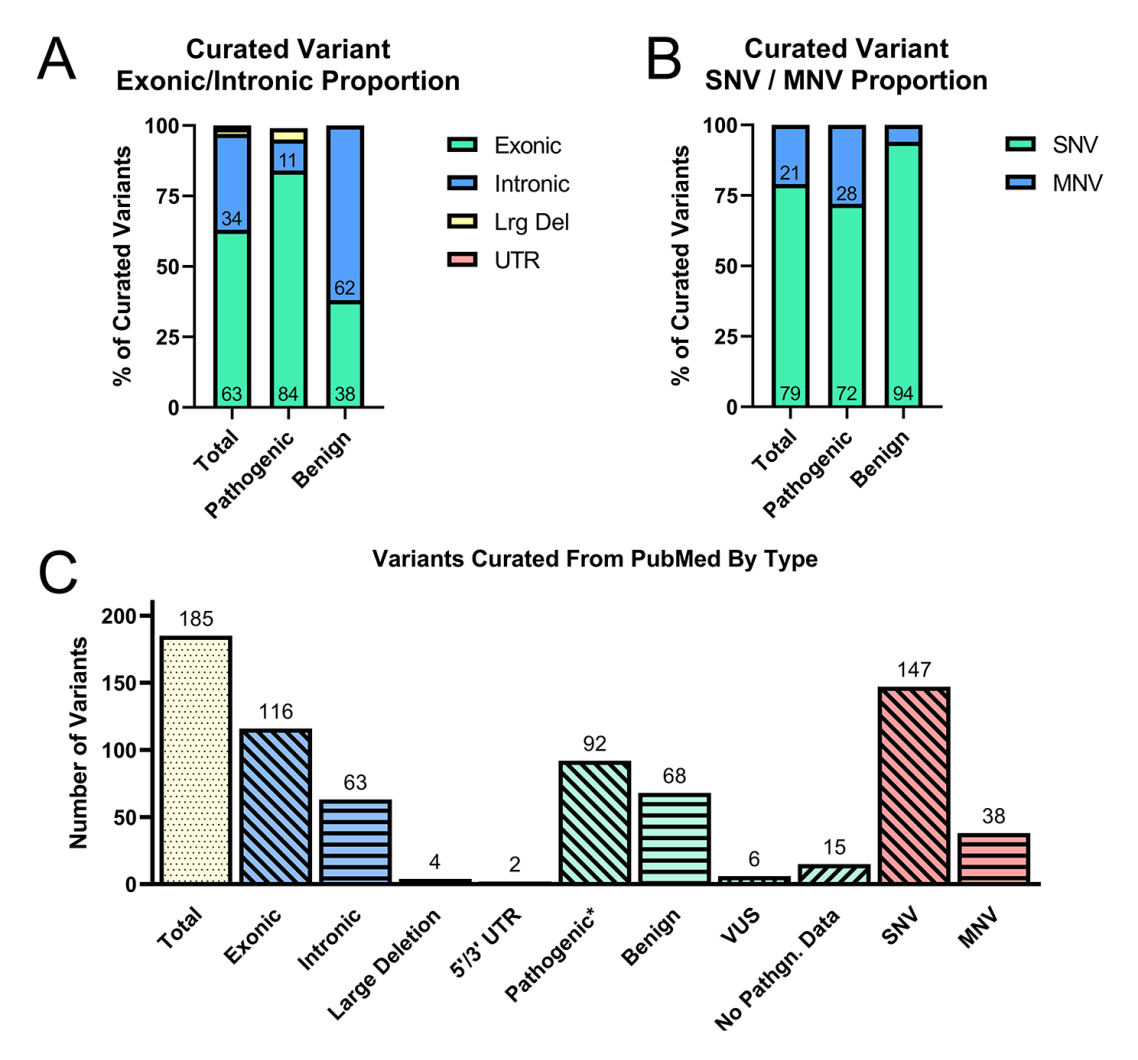
Curated *SLC6A8* Variants. Variants curated from PubMed publications are displayed by subtype. Percent curated variants that were (**A**) exonic, intronic, large deletions, or in the 5’ or 3’ untranslated regions (UTR) for total, pathogenic, and benign variants. The percent is shown inside the bar. (**B**) single nucleotide (SNV) or multi nucleotide (MNV) variants are shown. (**C**) The number of variants per subtype is shown. The majority of variants curated from PubMed were exonic SNVs. Pathogenic*: Pathogenic variants except large deletions

**Fig. 3:**
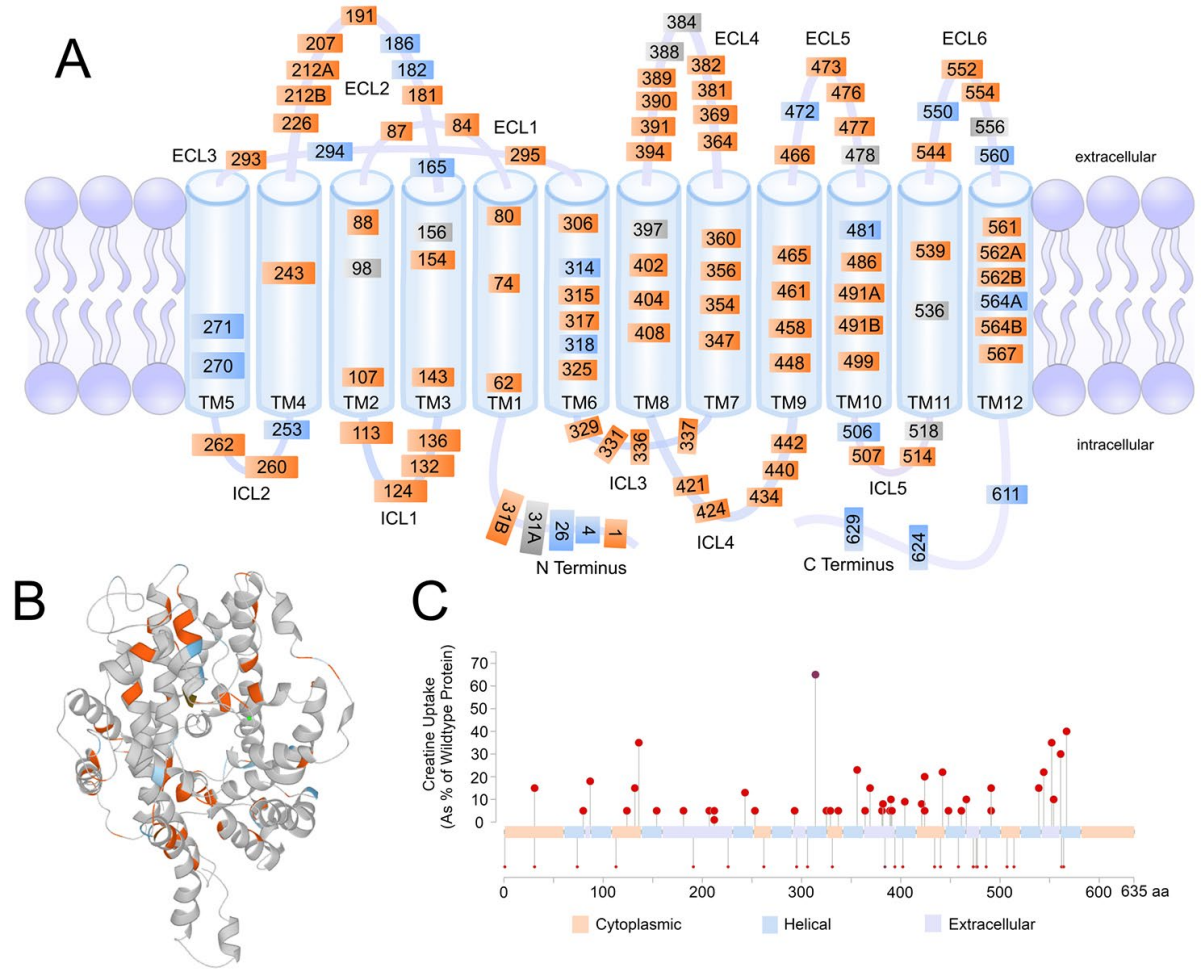
Variants in *SLC6A8*. The curated published single nucleotide exonic variant positions are shown on the 2-D (**A**) and 3-D (**B**) models of the structure of *SLC6A8*. The source for the 3-D model was AlphaFold, which was developed by DeepMind and EMBL-EBI. Orange: Likely Pathogenic and Pathogenic. Gray: Uncertain significance. Blue: Likely Benign and Benign. Full variant details can be found in the Supplemental Information. (**C**) Pathogenic variants are displayed as a lolliplot plot along the protein sequence; variants with available impaired creatine uptake rates are plotted above the line and variants with unmeasured creatine uptake are displayed below that line

Of the 185 unique variants, there were 4 large deletions where multiple exons or the entire *SLC6A8* gene was deleted. Of the 181 non-large-deletion variants, 63 were intronic (34%), 116 were exonic (63%) variants, and 2 were in 5’ or 3’ regions (1%). These 181 published variants included 92 classified as pathogenic or likely pathogenic (50%) for CTD, as determined by a clear clinical and functional association mentioned in the manuscript, 68 benign or likely benign (37%), and 21 variants (11%) of uncertain significance or without any evidence regarding their association with CTD. Of the total 185 variants, there were 147 single nucleotide (79%) and 38 multi nucleotide variants (21%), see Fig. 2. Figure 3 shows the curated variants plotted on the structure of *SLC6A8* in (A) 2-dimensions, (B) 3-dimensions, and (C) as a lollipop plot of pathogenic variants displaying impaired creatine uptake relative to wild type on the linear sequence. The source for the 3-D model was AlphaFold [42], which was developed by DeepMind and EMBL-EBI. The rate of variants mentioned as *de novo* (novel in the patient and not present in the parents) was 14% amongst the published variants.

Detailed analysis was performed on these variants with two questions in mind: (i) How likely is it for a rare disease researcher to find all known variants associated with the disease in a well-known publicly accessible database? (ii) How likely is it for a variant to be assigned the correct pathogenicity classification by an impact prediction algorithm?

### Validation of *SLC6A8* curated variants by comparison to LOVD

**Fig. 4:**
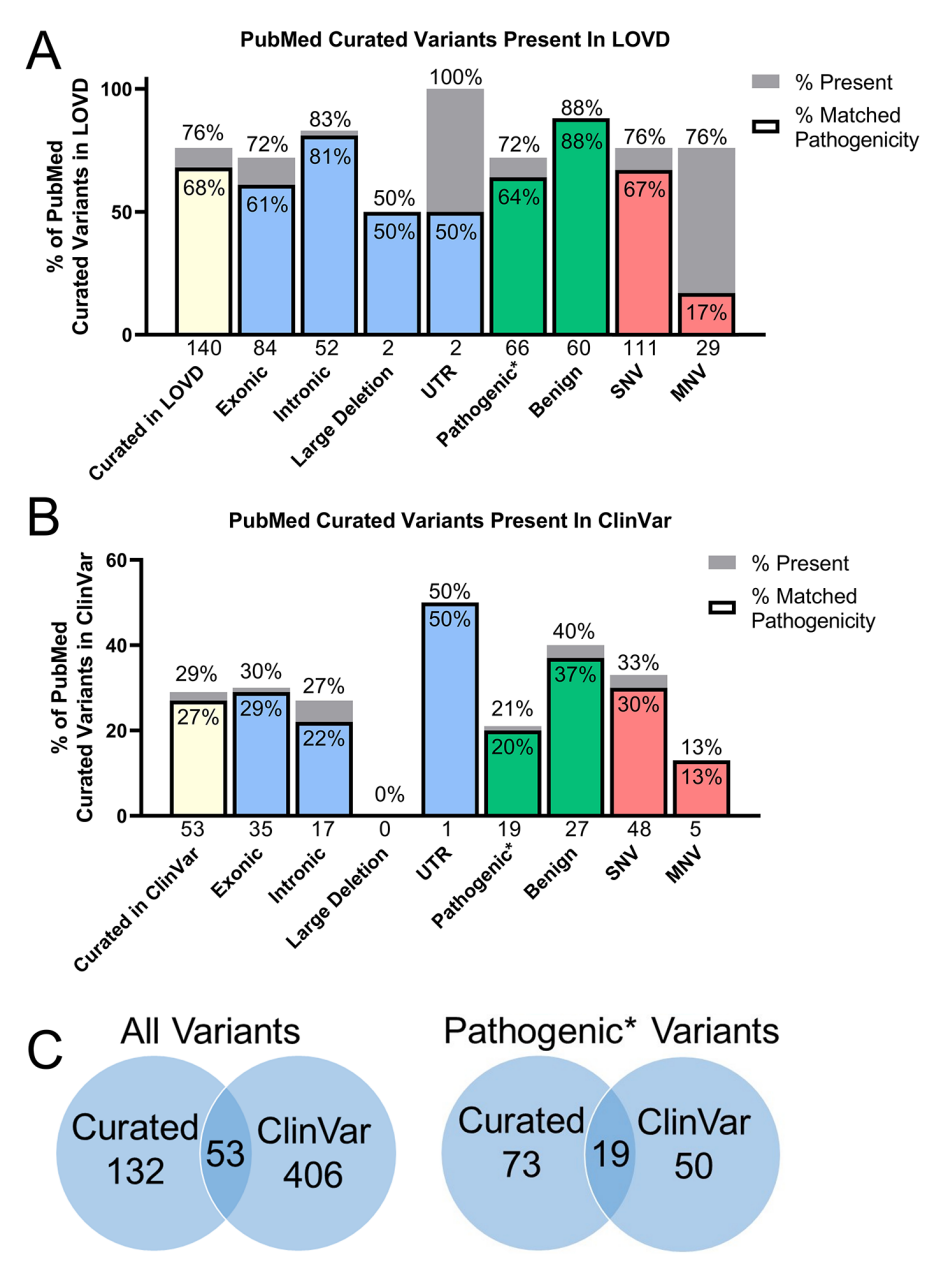
Curated Variant List Overlap with LOVD and ClinVar databases. *SLC6A8* variants curated from PubMed publications were compared with those recorded in LOVD (**A**) and ClinVar (**B**). Percent present (above bars) and total number (below x axis) of our curated list of 185 published variants are shown for total and by subtype. The percent within the bar shows the percent that matched the pathogenicity classification. (**C**) Overlap of total and pathogenic* variants in the curated list and ClinVar database. Only 53 of the 185 published variants were present in ClinVar. Pathogenic*: Pathogenic variants except large deletions

The accuracy of our methodology and results obtained were assessed before performing analysis. Similar to validating results by comparing to a reference standard or third party result, our curation process was validated by comparing the list of our curated variants to the list compiled in the LOVD [41] *SLC6A8* database. There were 183 *SLC6A8* variants in the LOVD database accessed April 2021. Of those, 140 were in our curated variant list, see Fig. 4A. Of the 43 variants that were in LOVD but not in our curated variant list, 6 were published but not identified by our curation process, while 37 were entered into LOVD via the contribution of researchers sharing their unpublished data. Of the six LOVD variants that were published but missed by our curation (4%), one was missed at the literature access step and five were missed in the variant curation step (two due to incomplete curation of a table inside the main manuscript and three were present in a supplemental attachment that was missed during curation).

An analysis of the data showed that LOVD had 76% of the variants that were in our curated variants list (Fig. 4A). The manual curation effort uncovered 45 variants not reported in the LOVD *SLC6A8* database. This was somewhat expected as the LOVD *SLC6A8* database is largely developed through submissions by individual labs, and not all researchers are aware of or have contributed their variant findings to this open-source database. LOVD had 88% of the benign and 72% of the published pathogenic *SLC6A8* variants.

### Comparing the curated variant list with ClinVar

Because our curated *SLC6A8* variants were of clinical relevance, comparisons were performed with the ClinVar database (Fig. 4). Our curated variant list contained all ClinVar variants that were reported as having been published. There were 459 *SLC6A8* variants in ClinVar as of Aug 2021 (Fig. 4C). ClinVar had 29% of the total variants curated from PubMed articles. Of these, 94% had the same pathogenicity rating in ClinVar and matched the curated pathogenicity rating. An example discrepancy is c.76G > A, p.G26R, HG38 chrX: NC_000023.11: 153,688,650 G > A, which is rated a VUS in ClinVar, but was rated benign in the curated dataset because of evidence that its creatine uptake was within 25% of wild type transport [43]. 21% of the pathogenic variants curated from PubMed published manuscripts were present in ClinVar. Figure 4B shows the percent of exonic, intronic, large deletion, untranslated region, non-large-deletion pathogenic, benign, SNV, and MNV curated variants that were present in ClinVar.

### Comparing the curated variant list with dbSNP, gnomAD, and 1,000 genomes

**Fig. 5:**
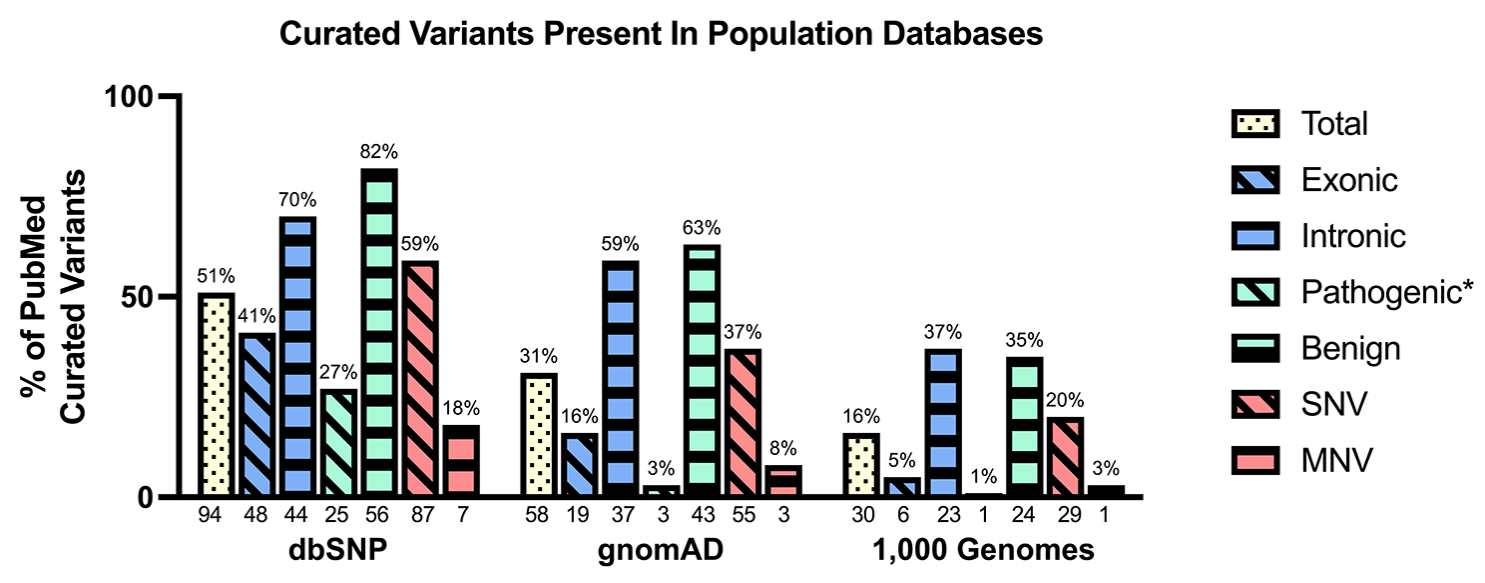
Curated *SLC6A8* Variants in Population Databases dbSNP, gnomAD, and 1,000 Genomes. Both percent (above bar) and number of variants (below x axis) are shown. Pathogenic*: Pathogenic variants except large deletions

Overlap with dbSNP [44] was assessed as it’s the largest database of researcher contributed single nucleotide polymorphisms, while gnomAD [45] and 1000 genomes [46] contain variants identified through whole genome or exome sequencing in large cohorts. CTD is an X-linked disorder and females can be asymptomatic carriers of pathogenic variants, meaning pathogenic variants could also be found in control populations. The comparisons also allowed analysis on minor allele frequencies in any variants uncovered through the large population sequencing projects. The variant type percentages for total, benign, and pathogenic PubMed published *SLC6A8* variants that were present in these databases are shown (Fig. 5). Comparison with the pathogenic variants in the curated data set to the public databases found that dbSNP, gnomAD, and 1,000 Genomes had 27%, 3%, and 1% of the pathogenic curated variants, respectively.

### Type of variant affects translation to databases

24% (22/92) of the pathogenic variants found in PubMed articles were not captured in any database used in this analysis, compared with 6% (4/68) of benign variants. 79% of the *SLC6A8* variants published in a scientific journal with strong clinical evidence for their pathogenicity were not present in the clinical association variant database ClinVar. The published multi-nucleotide variants in our data set were significantly less likely than SNVs (p = 0.03, unpaired t test with Mann-Whitney) to be included in ClinVar, 1,000 Genomes, gnomAD, and dbSNP.

### Accuracy of in-silico algorithm predictions of *SLC6A8* variant pathogenicity

**Fig. 6:**
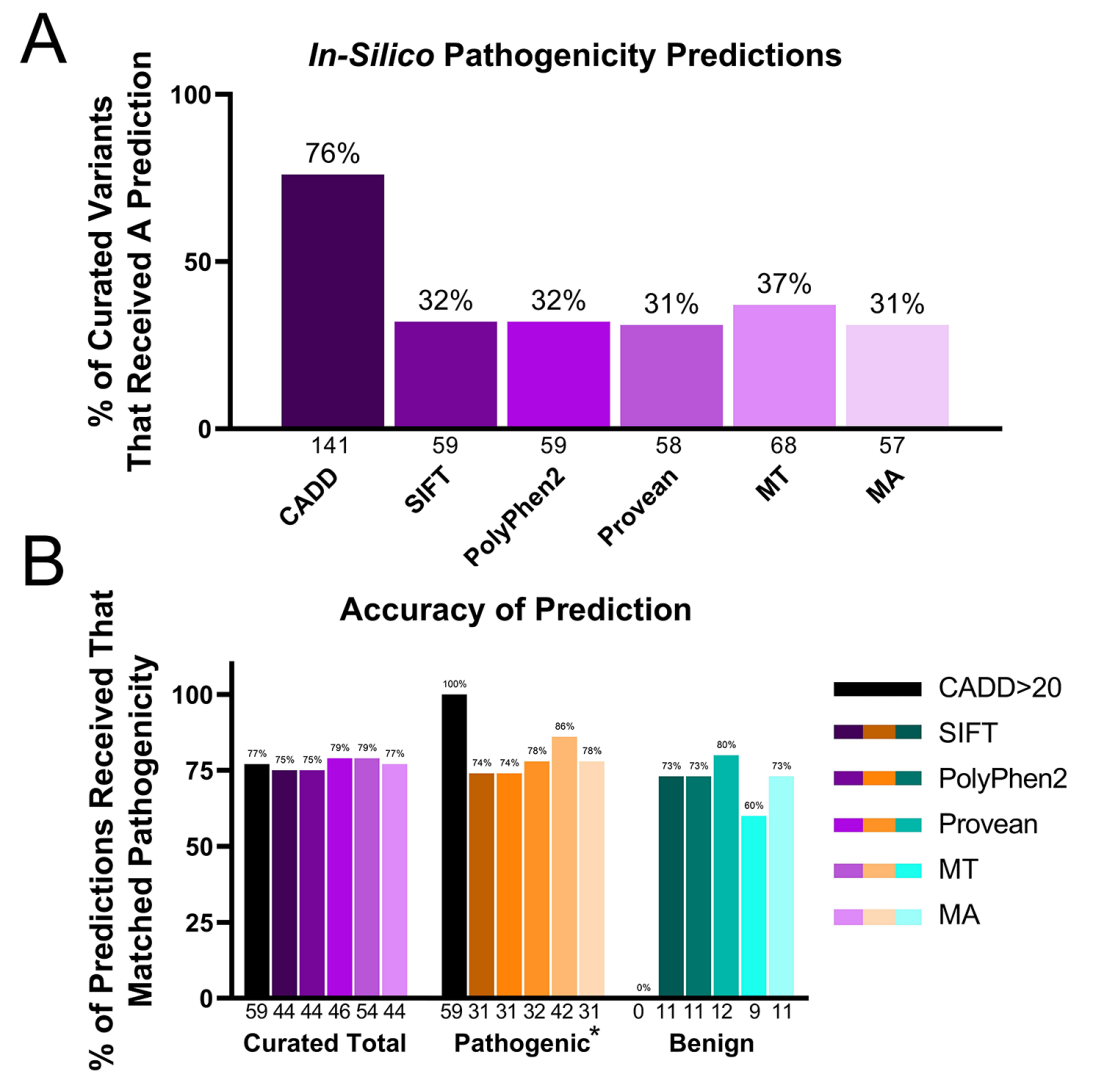
Curated *SLC6A8* Variants Impact Prediction. (**A**) The number of predictions made from the total number of curated variants and (**B**) the accuracy of the prediction for *in silico* pathogenicity predictors are shown. The percentage is above the bar and the number is below the axis. Pathogenic*: Pathogenic variants except large deletions

We analyzed the number of predictions generated by modeling algorithms for different types of variants (exonic, intronic, single nucleotide (SNV), and multi-nucleotide variants (MNV)). Next, we asked how many of these predictions correctly matched the pathogenicity rating in the curated variant list. Figure 6 shows the variant pathogenicity predictions from commonly used *in-silico* algorithms including SIFT [47], PolyPhen2 [48], MutationTaster2 [49], Mutation Assessor [50], and PROVEAN [51]. Impact predictions from these algorithms are mostly limited to coding regions, as the algorithms use protein sequences to assign functional impact. For this reason, CADD [52] scores for single nucleotide variants were also included in the analysis.

SIFT generated a prediction for 32% of the curated variants. Of these, it accurately predicted pathogenicity in 73% of pathogenic variants and 74% of benign variants that received a prediction. PolyPhen2 generated a prediction for 32% of the curated variants, accurately predicting pathogenicity for 74% of pathogenic and 73% of benign curated variants. MutationTaster2 generated predictions for 37% of the curated variants, predicting pathogenicity accurately for 86% of curated pathogenic variants that received a prediction and 60% of benign variants. PROVEAN made a prediction for 31% of the curated variants, accurately predicting pathogenicity for 78% of pathogenic variants and for 80% of benign variants.

CADD scores were obtained for all single nucleotide substitutions and the scores were available for 76% of the 185 total curated variants. 72% of the benign variants received a CADD score of less than 10 and were correctly rated benign. Using a cutoff of 20 [53], where a prediction of x < 20 is interpreted to mean benign and x > 20 is interpreted to mean pathogenic, CADD accurately predicted pathogenicity for 65% of the variants. The efficacy of a CADD cutoff of 20 was compared versus a cutoff of 10 or 30. Of the 91, 77, and 23 variants that received a CADD score of greater than 10, 20, and 30, respectively, 66%, 77%, and 90% had evidence of pathogenicity in patients. Of the four variants that received a CADD score of greater than 30 but were not categorized as pathogenic, one had 78% of wild type creatine uptake and was considered not impaired enough to be pathogenic, one was a frameshift closer to the N than C terminus but without sufficient published phenotype information to rank as pathogenic, one had no evidence for or against pathogenicity, and another was one of multiple variants in the patient.

65% of the 185 total curated variants received an accurate pathogenicity prediction from an algorithm. Of those accurate predictions, 54% were solely based on CADD scores. 30% of the curated variants did not receive an accurate prediction from any algorithm.

### Potential impact of curated variants on protein function

Sites of known or predicted protein structural features were identified and analyzed for overlap with the curated variants. The human *SLC6A8* gene has a cDNA length of 2,283 bp and encodes a protein of 635 amino acids that contains 12 putative hydrophobic transmembrane domains [54]. It is a Na^+^ and Cl^−^ dependent [55] transporter of creatine [56]. Two sites were noted in the literature to be potentially important in stabilizing alpha helices: G132 [57] which is the site of a pathogenic variant in patients, and Y148 [58], which has not been published as a variant occurring in patients. Amino acids predicted to be involved in creatine binding specificity included F68 [59], C144 [58], F314 [58], F315 [58], A318 [59], P382 [57], and G421 [59] – which are also pathogenic variants found in patients, and L72 [58], G73 [58], L321 [58], and S417 [58], which are locations for which variants have not yet been observed in patients. Sites predicted to impact phosphorylation [60] included S5, Y11, S12, S14, T618, T620, S623, and S625. None of the curated variants occurred at these predicted phosphorylation sites. None of the sites of glycosylation T171 [61], T175 [61], T178 [61], N192 [54], N197 [54], or N548 [54] were locations of published variants. Leucine zippers have been proposed for L286 [54], L293 [54], and L300 [54], none of which were locations of published variants. A disulfide bridge has been proposed for *SLC6A8* between C172 and C181 [57], and C181 is a site of a pathogenic patient variant.

For CTD, Salazar et al. [57] proposed six classes of variants for *SLC6A8* based on the type of transcription, translation, retention, folding, or functional disruption caused by the variant. These classifications can be used to cluster variants with the same mechanism of dysfunction, such as suspected misfolding, and investigate whether creatine transport could be restored to the multiple variants of this class by an intervention, such as the FDA approved chaperone protein 4-PBA [62]. Investigation of the impact of 4-PBA on creatine transport [57, 61] identified some variants for which 4-PBA increased creatine uptake (G337W, R391W, A404P, G424D, A448D, V539I, P544L, P554L) and some variants for which 4-PBA did not increase creatine uptake (Y80H, G87R, G132V, G253R, G356V, P382L, P390L, G421R, C491W), see Table 2. The classification of each variant based upon type of disruption is also annotated in the supplemental table.

**Table 2:** Effect of 4-PBA. Impact of 4-PBA on creatine uptake and glycosylation changes for *SLC6A8* variants compared to wild type

Variant	4-PBA Impact on Mature Glycosylation	4-PBA Impact on Creatine Uptake
p.Y80H	4-PBA increased mature glycosylation	Creatine uptake of 0% of WT was not increased after 4-PBA
p.G87R	4-PBA increased mature glycosylation	Creatine uptake of 0% of WT was not increased after 4-PBA
p.G132V	4-PBA did not increase mature glycosylation	Creatine uptake of 0% of WT was not increased after 4-PBA
p.G253R	4-PBA did not increase mature glycosylation	Creatine uptake of 0% of WT was not increased after 4-PBA
p.G337W	4-PBA did not increase mature glycosylation	Creatine uptake increased from 0–15% after 4-PBA
p.G356V	4-PBA did not increase mature glycosylation	Creatine uptake of 0% of WT was not increased after 4-PBA
p.P382L	4-PBA increased mature glycosylation	0% of wild type creatine uptake did not increase after 4-PBA
p.P390L	4-PBA increased mature glycosylation	Creatine uptake of 0% did not increase after 4-PBA
p.R391W	4-PBA increased mature glycosylation	Creatine uptake of < 10% increased to 30% of WT after 4-PBA
p.A404P	4-PBA increased mature glycosylation	Creatine uptake of < 10% increased to 30% of WT after 4-PBA
p.G421R	Not Measured	< 20% wild type creatine uptake not rescued by 4-PBA
p.G424D	4-PBA increased mature glycosylation	Creatine uptake of 0% of WT increased to 50% of WT after 4-PBA
p.A448D	4-PBA increased mature glycosylation	Creatine uptake increased from 0–10% of WT after 4-PBA
p.C491W	4-PBA increased mature glycosylation	Creatine uptake of 0% of WT was not increased after 4-PBA
p.V539I	4-PBA increased mature glycosylation	Creatine uptake increased from 10–50% of WT after 4-PBA
p.P544L	4-PBA increased mature glycosylation	Creatine uptake increased from 25–50% of WT after 4-PBA
p.P554L	4-PBA increased mature glycosylation	Creatine uptake increased from 0–50% of WT after 4-PBA

## Discussion

### Gaps in translation of published variants from PubMed to variant databases

24% of variants pathogenic for CTD were not known outside of published manuscripts. This result is likely not unique to this disease, but rather representative of the current sharing of knowledge for genetic variants for other rare diseases. The largest percentage of the published pathogenic variants for *SLC6A8* were found in the well curated LOVD3 variant resource specifically developed for this gene. However, our analysis showed that even when such gene specific resources are available, they might not contain all the pathogenic variants already identified and published in a literature article. Curated variant resources are not available for all rare diseases and the available variant details may be scattered across multiple information sources and hard to obtain. Currently, calls for research are focusing on disease-agnostic efforts [63] capable of being applied to all rare diseases. Text mining algorithms that retrieve variants from the literature could be applied to all rare diseases. Algorithms are scalable in a way that human curators are not, and once properly trained and tuned, a text mining algorithm could feasibly retrieve variants from the literature for all known [5] rare disease associated genes. This is a complex task requiring collaborations and crosstalk across multiple institutes. To aid in this process, all the variants and associated pathogenicity evidence on this one monogenic rare disease are shared in the supplemental table of this manuscript.

### Need for harmonizing variant notation

One of the challenges to this investigation were the multiple naming conventions used to name gene variants. 12% (25/210) of the initial curated variant list were consolidated as duplicates published under multiple names. Authors did not uniformly all publish using c. nomenclature, with some historical variants using IVS nomenclature. Intronic variants were named by both the + or – naming convention to denote the end or beginning of the nearby exon. There was no consensus on how to name variants with repeated bases, for example a variant (HG38 chrX: NC_000023.11: 153,693,407 dup TGCCC) being published as both c.1016 + 41_45dupTGCCC and c.1016 + 41dupTGCCC. Single amino acid nomenclature was inaccurately assigned especially regarding the residues K, L, D, and N [64, 65]. It would reduce inaccuracies to publish variants using three letter amino acid nomenclature such as Lys, Leu, Asp, and Asn when possible, rather than single letter notation. Stating the protein’s amino acid change alone is insufficient for reporting genetic variants because there are situations where the amino acid could have been the result of multiple possible codons. Synonymous variants can cause disease [66]. It should therefore be standard to clarify the genetic sequence change, as well as reporting the protein sequence variant name. There was a variant that was published under the notation c.1151-8 C > T [67] with evidence that included trio segregation sequencing, symptoms, and decreased MRS measured brain creatine supporting a pathogenic classification, but was actually located at c.1496-8 C > T (HG38 chrX: NC_000023.11: 153,694,525 C > T) as notated by Betsalel 2011 [68], Betsalel 2012 [64], and Cameron 2017 [69] who *used in-silico* prediction algorithms and classified the variant as likely benign. Any researcher who had not reviewed and mapped the published sequence from Jiang 2018 [67] to the right genomic positions would not have known that the *in-silico* predicted likely-benign c.1496-8 C > T variant has strong clinical evidence supporting a pathogenic classification under the notation c.1151-8 C > T. Researchers also lacked consensus on which was the canonical protein accession number to reference, for example the variant HG38 chrX: NC_000023.11: 153,694,347 G > A being published under the names p.Gly466Arg and p.Gly351Arg depending upon choice of protein reference. This example demonstrates why publishing the protein accession number is useful when referring to a variant written in protein notation. The harmonization process and the difficulties associated with consolidating multiple variant nomenclatures highlighted the importance of including Human Genome Variation Society (HGVS) position notation for all published variants (https://varnomen.hgvs.org/bg-material/standards/). An automated workflow would need to harmonize variants to genomic position to avoid ambiguity and accurately consolidate variants with repeated names to one entry.

### Pathogenicity predictions remain imperfect

As genome sequencing data becomes more available, there is a greater need to interpret the functional classification of novel variants of uncertain significance, both intronic and exonic, identified in rare disease patients [70]. We are currently limited in our ability to predict which intronic variants cause disease [71], as most impact prediction algorithms are trained with protein sequences and are limited to coding regions. Algorithms such as CADD can generate functional impact scores for SNVs in any area of the genome, as seen by pathogenicity prediction scores by CADD for 87% of the intronic variants. The majority (88%) of the 92 curated pathogenic variants were exonic. The major source of failure to assign the correct pathogenicity prediction was not receiving a prediction; only 31 to 76% of total variants received a prediction from the various *in-situ* predictor algorithms (Fig. 6). Prediction failures occurred when the variant was intronic, synonymous, not a SNV or because the coding region for that variant isn’t well conserved between species, as many of these algorithms employ sequence homology to determine pathogenicity. Of the variants that received a prediction, between 75 and 79% were accurately predicted to be pathogenic by *in-silico* prediction algorithms PROVEAN, PP2, SIFT, MA, MT, and CADD.

Our findings that fewer than 60% of the published exonic pathogenic variants were correctly predicted to be pathogenic by any single pathogenicity predictor confirms findings from other studies [72] that these algorithms have not yet reached clinical reliability to classify the pathogenicity of novel variants discovered in patients. Assumptions and limitations of the protein function prediction algorithms stresses the importance of reporting variant protein activity relative to wild type protein activity whenever assays are available to the researchers and clinicians. Large scale data on protein activity relative to wild type function could potentially be used to improve future *in-silico* predictor models.

### Possible novel variants in other populations

Most of the variants curated in this effort were contributed by research groups based in Europe. It is important that future sequencing efforts be supported at different geographical locations as population-based differences will likely uncover novel pathogenic *SLC6A8* variants in patients.

### Scaling the effort

Identifying the pathogenic genetic variant is important not only to the individual patient with the rare disease, but also to future patients and to researchers seeking to understand the protein’s structure-function relationship. However, rare disease researchers, clinicians, and patients may find themselves siloed alone in their immediate circle of contacts, unaware of data repositories where they could share their discoveries with fellow members of the rare disease community. Sharing information worldwide to prevent repeated effort is critical. An example failure of communication of discoveries would be if one clinical research group worked to classify a variant as pathogenic but then didn’t know where to share the information, and later a second group expended effort to classify the same variant already known to the first or to several other siloed groups. One attainable goal is for information on classified variants to be better disseminated. With more than 3,000 rare diseases with known gene associations [5], and each disease having hundreds of publications, the task of manually curating literature for each of these rare diseases and keeping that curation up to date is infeasible. Automatically curating or text mining the published literature to retrieve all variants and their phenotypes as they are published could help disseminate the missed variant details found in published manuscripts but not present in any publicly accessible databases. Manual curation of phenotype-genotype relationships is essential for the production of high-quality databases, but it is a costly and time-consuming process. Fully automatic solutions would be needed in order to efficiently and cost-effectively address the scale of identifying these relationships within biomedical literature [73].

Utilization of Biomedical Natural Language Processing (BioNLP) and text-mining techniques could allow for the automatic extraction of critical information found in biomedical literature, including genetic diseases and the associated variants [74, 75]. One attempt to extract triplets of disease-gene-variants from biomedical literature utilized machine learning tools such as GNormPlus [76], tmVar [77], and DNorm [78] to extract the entities of diseases, genes, and variants [74]. These tools employ algorithms such as the Conditional Random Field (CRF) model, which is specifically designed for sequence labeling tasks such as Named Entity Recognition (NER). The CRF model is trained on a dataset of entities that are annotated for each type, allowing for accurate identification and labeling of named entities in text. This is followed by a normalization step, which harmonizes the identified entities to a common nomenclature. This normalization step ensures consistency, disambiguation, and linking to external knowledge bases, while also improving the performance of downstream applications through more accurate and consistent information extraction. In another study, deep learning extracted variant-gene-drug relationships from the literature [79]. The authors used two computational methods to extract gene-mutation-drug relations from biomedical literature. The first method uses the Biomedical Entity Search Tool (BEST) scoring results as features in a machine learning classifier. The second method uses BEST scoring results and word vectors in a deep convolutional neural network model. These methods are able to extract variant-gene and variant-drug relations from literature using machine learning classifiers like random forest and deep convolutional neural networks. Transformer models, specifically the Bidirectional Encoder Representations from Transformers (BERT) model, have revolutionized the field of natural language processing (NLP) and have become the state of the art technique for a wide range of NLP tasks. These methods have excelled in the tasks of named entity recognition to extract the diseases, genes, and variants, relation extraction, document multilabel classification, and inference tasks [80]. Finally, recent advances in transformer-based models have allowed for the development of domain-specific generative language models that are pre-trained on large datasets of biomedical literature such as BioGPT [81]. These models outperformed previous models on tasks such as relation extraction, question answering, and document classification.

Although the accuracy of these algorithms is not close to manual curation, they do provide a mechanism for mining thousands of articles in a timely manner. The biggest impediments to the current automated variant mining efforts from literature is the ability to map the variants to the correct gene and assign the right pathogenicity categories by capturing context details. Based on our analysis and gaps identified, we propose that text mining efforts incorporate variant harmonization into their workflows and ensure that the base or amino acid referred in a variant notation is verified by validating with reference sequences for the specific gene and/or protein. In addition, the context details captured through the curation effort, also showed the importance of tailoring literature curation workflows for each rare disease and gene combination. For example, clinical phenotypes and creatine uptake ratios mentioned in the articles were used to assign pathogenicity tiers in our manual curation effort and will be equally important for any automated text mining efforts.

For automated systems, it may be possible to design a multi-step approach for assigning pathogenicity predictions from large scale text mining efforts rather than being limited to relying on impact prediction algorithms alone. The overlap of pathogenicity predictions from multiple algorithms or an aggregate assessor such as REVEL [82] can be used as a first step for assigning pathogenicity for those variants that have multiple algorithms agreeing on the impact assignment. A second level of assignment could rely on algorithms such as CADD and use a high cut-off for assigning pathogenicity. A third level of critical analysis and context mining might be required for those variants where pathogenicity could not be assigned at the first or second levels. It will be important to mine for disease specific phenotypic details as well as gene and disease specific functional or biochemical assay details for these variants.

The information curated from literature during this study was specially tailored for rare disease variant extraction and pathogenicity classification. To help improve text mining workflows, we collected all the variants, clinical symptoms, and other functional details for each of the variants mentioned in a manuscript. This curated variant list for *SLC6A8* can serve as a training or validation data set of known accuracy, coverage, and genotype-phenotype associations. Enhanced text mining will significantly decrease the time necessary to gather data to molecularly characterize a rare disease and render it possible to mine rare disease phenotype-genotype associations for the thousands of rare disease genes [5] in a timely fashion.

## Conclusions

*SLC6A8* was chosen as a rare disease gene for this study as it has a well understood X-linked hemizygous inheritance and clear relationship between creatine transporter protein function and CTD disease phenotype. We curated hundreds of published manuscripts available through PubMed documenting *SLC6A8* gene variants in CTD patients and found 181 non-large-deletion variants for *SLC6A8* in PubMed, of which 92 were classified as pathogenic or likely pathogenic for CTD, as determined by a clear clinical and functional association mentioned in the manuscript.

This study investigated the translation of information about rare disease genetic variants published in PubMed accessible journals to open-source databases. We found that for one rare disease gene, 24% of the variants published in PubMed were not in any open access databases. Pathogenicity prediction algorithms made a prediction for fewer than 60% of published pathogenic variants. Manual curation of variants from literature is time consuming and developing text mining workflows by integrating the current state of the art natural language processing methods with impact prediction algorithms, disease phenotypes and functional assay details will help scale the effort to all rare genetic diseases.

While text mining might be the only option for finding variants from already published literature, it is also imperative that information on new variants discovered be available in structured databases. The genetic disease field will benefit from a streamlined dual submission process or requirement to submit pathogenic variants to a publicly maintained database such as NCBI’s ClinVar and use HGVS standardized variant notations in published literature. As the field of text mining develops innovative methods to extract variant information from published literature, it may become possible to use automated text mining algorithms to populate gene variant databases with pathogenic variant information as soon as it is published. Today’s authors can use web tools such as PubReCheck [83] to confirm that their manuscript is readable by text mining algorithms. Variant information is important for diagnosis, research, and treatment. Therefore, contributing variant information to the public databases and using standard variant notation will ensure that important gene disease associations are easily accessible.

As a future direction, we intend to continue the curation process, both by manually curating variants associated with other rare diseases, and by contributing to the text mining efforts in the rare disease space. The genotype and phenotype information curated as a dataset by this study is made available in the public domain for any researchers working on text mining algorithms. We also aim to integrate details from disparate data sources and better understand genotype-phenotype correlations in rare diseases, facilitating research inquiries that lead to further investigations, with the larger goal of improving diagnoses and therapeutic interventions for rare disease patients.

## Methods

**Fig. 7:**
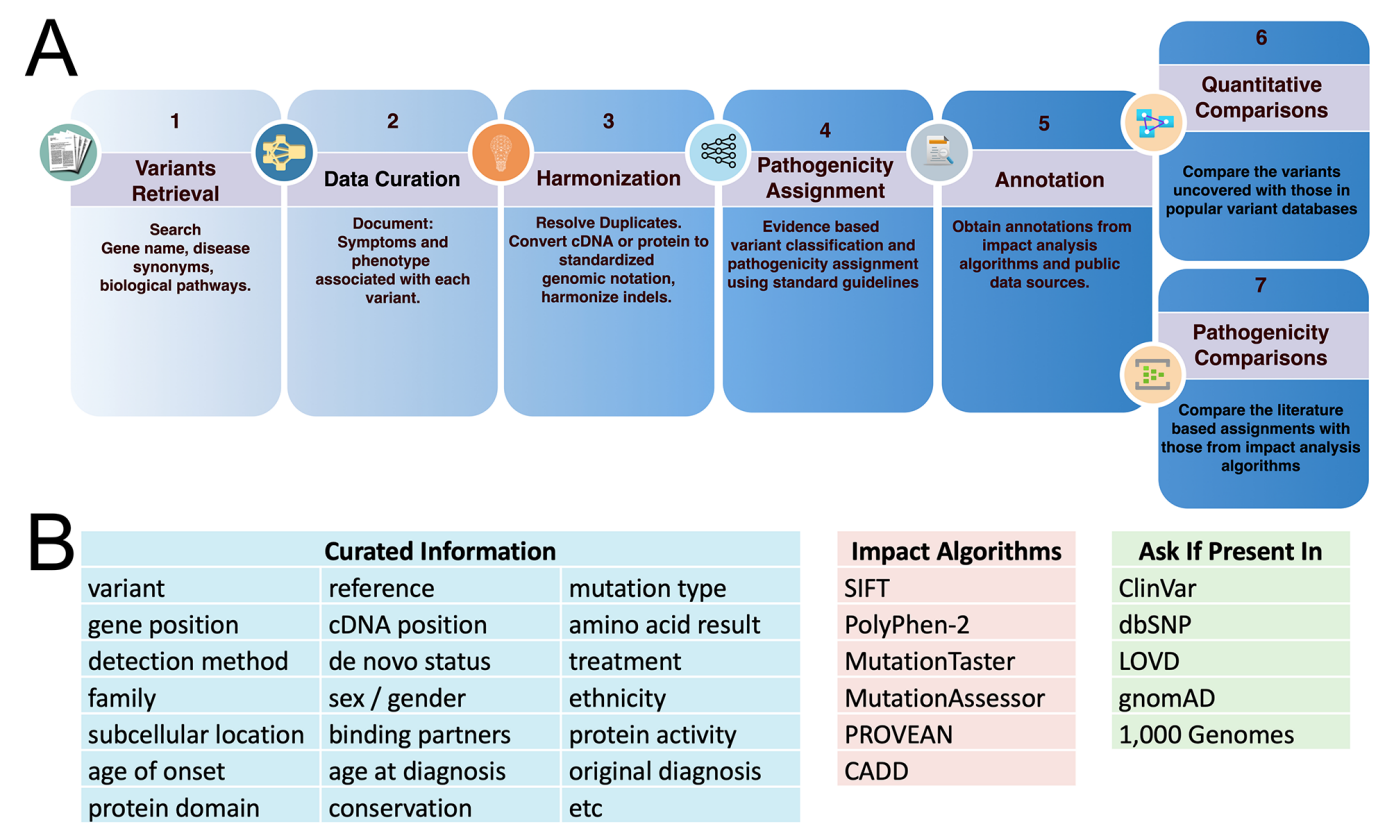
Methodology Workflow. (**A**) The curator discovered published rare disease genetic variants by searching PubMed for the gene name, disease synonyms, and biological pathways. Variants were compiled into a spreadsheet that included symptoms and phenotype associated with each variant and details that informed a pathogenicity assignment. The cDNA or protein variant names were harmonized to standard genetic notation. Impact analysis comparing pathogenicity predictions from algorithms to the ones reported in literature and comparisons with public data sources for finding the overlap and gaps of published variants were performed. (**B**) The categories of information collected and impact analysis algorithms and databases consulted. The full information is available as supplemental data

The workflow for curating the variants and assigning pathogenicity is depicted in Fig. 7A. All known published variants associated with CTD were obtained through the following steps (i) **Variant Retrieval**: Search published literature for variants in genes associated with CTD, (ii) **Data Curation**: Review, cross reference and document all phenotypic, clinical, and protein function details for the variants identified in any published literature, (iii) **Harmonization**: Standardize variant notations from all curated variants to remove ambiguity, map to a standard human reference genome and consolidate duplicate names, (iv) **Pathogenicity Classification**: Review the clinical and functional detail provided to assign pathogenicity categories to each variant based on the ACMG standards [84], (v) **Annotation**: Obtain annotations for all variants from public variant databases and predictions from impact analysis algorithms. Categories of curated information, impact analysis algorithms, and databases used in the analysis are shown in Fig. 7B.

### Variant retrieval

Our manual curation process for this one rare disease, CTD, involved reading more than two hundred peer reviewed manuscripts indexed in MEDLINE and PubMed with the goal of finding all published variants and phenotypes for the known disease gene *SLC6A8*. Multiple searches were performed in PubMed using the disease name (creatine transport deficiency), disease synonyms (CTD, creatine transport disorder, X-linked creatine deficiency), gene symbols and biological terms of relevance (creatine transporter, CRTR, CT1, *SLC6A8*). A review was conducted to ascertain the relevance of the articles in the search results. Relevant citations were selected for in depth investigation. Through these iterative searches, more than 200 peer reviewed manuscripts including reports of individual patients, articles on protein structure modeling, the impact of genetic variants on signaling pathways, and reviews dating from 1975 to 2020 were retrieved. All variants, reported using both HGVS nomenclature and a variety of non-HGVS nomenclatures, were recorded.

### Data curation

A spreadsheet list of all *SLC6A8* variants was created that cited the reference in which the variant was mentioned and recorded the variant, clinical symptoms, phenotype, patient details, and relevant functional assay results. The information extracted for each variant is detailed in the curated information category of Fig. 7B. Example terms captured in the symptoms and test result categories for CTD include developmental delay, mental disability, hypotonia, behavioral problems, motor dysfunction, social smile, seizures, cerebral atrophy, creatine to creatinine ratio, delayed language acquisition, creatine peak, magnetic resonance spectroscopy (MRS), and apraxia. All variant notations used to represent the variant in the manuscript including any cDNA, protein, genic and genomic location references were captured. A total of 210 variants were obtained after the curation step. The complete information collected for these variants is available in the supplemental table.

### Harmonization

During the harmonization step, all notations of the variant were converted to standardized genomic notation using a custom script. The results were then manually validated and variants that could not be harmonized with the script were further analyzed and harmonized by comparing the genomic positions manually. In general, single nucleotide variants were harmonized with minimal issues, while indels were more difficult to convert to genomic notation.

After data curation, there were 210 curated variants, but harmonization revealed that 12% of these were duplicates of the same genomic alteration published under different nomenclatures. The harmonization step removed duplicates and reduced the variants obtained to 185, which included 4 large multi exon or multi gene deletions.

### Pathogenicity classification

We followed the American College of Medical Genetics (ACMG) guidelines [84] to classify pathogenicity based upon clear clinical and functional consequences for the variant from the literature source. The evidence used for pathogenicity classification included a lower cerebral creatine peak as measured by MRS, an elevated urine or plasma creatine to creatinine ratio relative to age matched controls, DHPLC or fibroblast creatine uptake, D3 labeled creatine wash out assay, and an in vitro measured impaired ability of the protein to transport creatine relative to measurements of the wild-type protein. Variants with clear clinical evidence or functional evidence of less than 70% creatine uptake activity were considered pathogenic. Variants were classified as benign if they were reported in males without CTD, did not have an inheritance that segregated with phenotype, or if the variant protein was shown to have close to wild type functionality. When conflicting evidence was reported in different manuscripts, they were classified as variants of uncertain significance. The curation process also uncovered reports of variants without any clinical or functional evidence for CTD. All such variants were not assigned a pathogenicity category. Table 3 shows example variants in each category and types of evidence used for their categorization.

**Table 3:** Assigning Pathogenicity. A lower MRS measured cerebral creatine peak, elevated plasma creatine to creatinine ratio, and impaired ability of the protein to transport creatine relative to the wildtype protein were all used as evidence of variant pathogenicity. The table lists a selection of variants from different categories, evidence found in the manuscript and the pathogenicity assignment made based on these details

Variant	Evidence in the manuscript	ACMG category
c.619 C > T, p.R207W	Patient had mental disability, increased urine Cr/Crn ratio, and the fibroblasts cultivated from patient cells had less than 10% of wild type creatine uptake.	Pathogenic
c.942 C > G, p.F314L	65% of wild type activity, close to the cutoff for residual activity.	Likely Pathogenic
c.1162G > A, p.A388T	Reported non-pathogenic by Betsalel 2011 but pathogenic by Cameron 2017. None of the referenced articles have data for brain MRS creatine peak, plasma creatine, urine creatine to creatinine ratio, or fibroblast creatine uptake rate compared to wild type protein.	Variant of Uncertain Significance
c.544G > A, p.V182M	Detected in a CTD patient, but the authors found that the variant did not segregate with phenotype in the family.	Likely Benign
c.394 + 88G > C	Detected in 21 of 166 non-CTD individuals.	Benign
p.T481I	Fibroblast creatine transport rate within 25% of the wild type protein’s creatine transport rate.	Benign

### Annotation

Annotations were performed on the 181 variants, after excluding the 4 large deletions, using AVIA [85] and VEP [86] applications. Variant annotations from multiple databases such as ClinVar [13], dbSNP [44], gnomAD [45] and 1,000 Genomes [46] were obtained, along with predictions from multiple variant impact analysis algorithms including SIFT [47], PolyPhen2 [48], MutationTaster2 [49], Mutation Assessor [50], PROVEAN [51], and CADD [52]. DisGeNet [87] and HGMD [88], two resources that include variants mined from literature, were not used for the variant comparisons as they only had a small representation of the curated variants in the public version.

### Electronic supplementary material

Below is the link to the electronic supplementary material.

**Additional file Table 1**.: Curated Variants and their analysis. This supplemental table contains all of the variants curated for this manuscript and their analysis

**Additional file Table 2**.: Supplemental Table of Genoox Classification of Curated Variants as Requested by the Reviewer. This supplemental table contains the Genoox classification and analysis

## Data Availability

We have submitted the full data as supplemental material. The PubMed curated variants list analyzed during the current study have been uploaded and are available in the LOVD repository, link: https://databases.lovd.nl/shared/genes/SLC6A8 .

## Abbreviations

4-PBA: 4-PhenylButyric Acid
ACMG: American College of Medical Genetics
AVIA: Annotation, Visualization, and Impact Analysis
BERT: Bidirectional Encoder Representations from Transformers
BEST: Biomedical Entity Search Tool
BioNLP: Biomedical Natural Language Processing
CADD: Combined Annotation Dependent Depletion
CTD: X-linked Creatine Transporter Deficiency
CRF: Conditional Random Field
dbSNP: Single Nucleotide Polymorphism Database
FDA: Food and Drug Administration
GAMT: Guanidinoacetate methyltransferase
GARD: Genetic and Rare Diseases Information Center
gnomAD: Genome Aggregation Database
HGMD: Human Gene Mutation Database
HGVS: Human Genome Variation Society
IVS: Intervening Sequence
LOVD: Leiden Open Variation Database
MNV: Multi Nucleotide Variant
MRS: Magnetic Resonance Spectroscopy
NLP: Natural Language Processing
NORD: National Organization for Rare Disorders
PolyPhen2: Polymorphism Phenotyping v2
PROVEAN: Protein Variation Effect Analyzer
SIFT: Sorting Intolerant From Tolerant
SNV: Single Nucleotide Variant
VEP: Variant Effect Predictor
VUS: Variants of Uncertain Significance
